# Challenges in Dengue Fever in the Elderly: Atypical Presentation and Risk of Severe Dengue and Hospita-Acquired Infection

**DOI:** 10.1371/journal.pntd.0002777

**Published:** 2014-04-03

**Authors:** Emily K. Rowe, Yee-Sin Leo, Joshua G. X. Wong, Tun-Linn Thein, Victor C. Gan, Linda K. Lee, David C. Lye

**Affiliations:** 1 Department of Infectious Diseases, Tan Tock Seng Hospital, Singapore; 2 Communicable Disease Centre, Institute of Infectious Diseases and Epidemiology, Singapore; 3 Yong Loo Lin School of Medicine, National University of Singapore, Singapore; Hospital for Tropical Diseases, Viet Nam

## Abstract

**Background/methods:**

To better understand dengue fever in the elderly, we compared clinical features, World Health Organization (WHO) dengue classification and outcomes between adult (<60) and elderly (≥60) dengue patients. We explored the impact of co-morbidity and hospital-acquired infection (HAI) on clinical outcomes in the elderly. All patients managed at the Communicable Disease Centre, Singapore, between 2005 and 2008 with positive dengue polymerase chain reaction (PCR) or who fulfilled WHO 1997 or 2009 probable dengue criteria with positive dengue IgM were included.

**Results:**

Of the 6989 cases, 295 (4.4%) were elderly. PCR was positive in 29%. The elderly suffered more severe disease with more dengue haemorrhagic fever (DHF) (29.2% vs. 21.4%) and severe dengue (SD) (20.3% vs. 14.6%) (p<0.05). Classic dengue symptoms were more common in the adult group. The elderly were less likely to fulfill WHO 1997 (93.6% vs. 96.4%) (p = 0.014), but not WHO 2009 probable dengue (75.3% vs. 71.5%). Time to dengue diagnosis was similar. There was no significant difference in the frequency of warning signs between the two groups, but the elderly were more likely to have hepatomegaly (p = 0.006) and malaise/lethargy (p = 0.033) while the adults had significantly more mucosal bleeding (p<0.001). Intensive care admission occurred in 15 and death in three, with no age difference. Notably, the elderly stayed in hospital longer (median 5 vs. 4 days), and suffered more pneumonia (3.8% vs. 0.7%) and urinary infection (1.9% vs. 0.3%) (p = 0.003). Predictors of excess length of stay were age (adjusted odds ratio [aOR] 2.01, 95% confidence interval [CI] 1.37–2.88), critical illness (aOR 5.13, 95%CI 2.59–9.75), HAI (aOR 12.06, 95%CI 7.39–19.9), Charlson score (aOR 6.9, 95%CI 2.02–22.56) and severe dengue (DHF/dengue shock syndrome/SD) (aOR 2.24, 95%CI 1.83–2.74).

**Conclusion:**

Elderly dengue patients present atypically and are at higher risk of DHF, SD and HAI. Aside from dengue severity, age, co-morbidity and HAI were associated with longer hospital stay.

## Author Summary

Dengue is a neglected tropical disease that is increasingly affecting elderly patients; however, there is a paucity of data on clinical presentation and outcomes in this group. The limited data suggests that elderly dengue patients have the highest case-fatality rate but the pathogenesis of mortality in elderly dengue patients remains unclear. To better understand dengue fever in the elderly we compared clinical features, WHO dengue classification and outcomes between adult (<60) and elderly (≥60) dengue patients and explored the impact of co-morbidity and HAI on clinical outcomes in the elderly. We found that diagnosis in the elderly may be challenging due to atypical presentation. Elderly patients have worse outcomes compared with their younger counterparts with increased rates of DHF and SD. Elderly patients have higher rates of HAI placing them at risk of infection-related mortality. Aside from dengue severity, age, co-morbidity and HAI were associated with longer hospital stay. This will place further burden on already stretched hospital systems.

## Introduction

Dengue is the most significant mosquito-borne virus in humans [1] and is endemic to Singapore. In Asia dengue classically affects children with the majority of cases of dengue hemorrhagic fever (DHF), dengue shock syndrome (DSS), and dengue related mortality observed in this group [2]. However, in Singapore dengue predominantly affects young adults possibly as a result of lowered herd immunity and acquisition outside of the home [3]. However, with aging population there has been an increase in dengue incidence rates in older adults [4], [5], [6]. In Taiwan older adults have the highest reported dengue incidence rate and risk of fatality [7]. Likewise in Singapore elderly patients accounted disproportionately for the majority of dengue deaths [8] highlighting the urgent need for enhanced understanding of dengue in the elderly to improve clinical management and outcome.

The cornerstone of management of dengue patients and prevention of dengue-related mortality is early diagnosis and recognition of clinical syndromes requiring intervention [9]. However diagnosis and appropriate management of elderly dengue patients may be delayed as they present atypically [10]. Fever may be the only symptom and leukopenia occurs less frequently compared with younger adults. Both World Health Organization (WHO) 1997 [11] and 2009 [9] dengue classifications have reduced sensitivity in older adults, because of the absence of classic dengue symptoms, potentially delaying diagnosis [12]. The atypical presentation is likely due to age-related decline in immune function, predominantly affecting cell-mediated and humoral immunity resulting in impaired cytokine response altering disease presentation [13].

Severe hospital-acquired infection (HAI) may contribute to dengue deaths in adults [14], [15]. Concurrent bacteremia was more common in elderly DHF patients (17.4%) compared with non-elderly DHF patients (3.4%) in Taiwan [16], but there was no significant difference in bloodstream infection rates between the fatal and non-fatal cases [16]. The impact of HAI in elderly dengue patients requires further investigation.

The limited data on elderly dengue patients suggests that this group has the highest case-fatality rate [7], [8], [17]. The pathogenesis of mortality in elderly dengue patients remains unclear but co-morbidities may play a role. Seventy-five percent of dengue fatalities in Singapore had co-morbidities [8]. Likewise, in Taiwan fatality from dengue was associated with age above 55 years and pre-existing hypertension, chronic renal impairment or diabetes [18]. In elderly patients with DHF pre-existing pulmonary disease and the development of DSS or acute renal failure (ARF) were associated with mortality in Taiwan [16]. In the 2002 dengue outbreak in Taiwan renal failure was both a risk factor for DHF and mortality, with the degree of renal impairment correlating to risk of mortality [19].

The aim of our study was to compare clinical features, WHO 1997 [11] and 2009 [9] dengue classification and outcomes between adult (<60 years of age) and elderly (≥60 years of age) dengue patients and explore the impact of co-morbidity and HAI on clinical outcomes in the elderly.

## Methods

### Ethics statement

The study was approved by the National Healthcare Group Domain Specific Review Board (DSRB/E/2008/00567) with a waiver of informed consent for the collection of anonymized data.

### Patients

A retrospective study of all adult dengue patients managed at the Communicable Disease Center (CDC), Tan Tock Seng Hospital was conducted between 1 January 2005 and 31 December 2008. Patients were hospitalized if they had suspected DHF or if they met previously published admission criteria [20]. Intensive care unit (ICU) referral criteria included patients with compensated shock (systolic blood pressure [BP] >90 mmHg but narrow pulse pressure <20 mmHg) and those with hypotension (systolic BP <90 mmHg). Patients who were not admitted had daily clinical assessment and full blood count until clinically stable.

All patients were managed using a standardized dengue care path improving consistency of clinical, laboratory, treatment and outcome data. The care path included daily documentation of symptoms (abdominal pain, bleeding, breathlessness and vomiting), examination findings (BP, rash, pleural effusions, ascites) and laboratory parameters (platelet count and hematocrit). The care path provided clear criterion for intravenous fluids and blood products. Medical interventions, diagnosis and patient outcomes (dengue fever, severe dengue [SD], DHF, DSS, severe bleeding, severe organ involvement) were documented daily within the care path. The hospital electronic medical records were used to extract laboratory, microbiological and radiological data. Data extraction was performed by medically trained research assistants.

Inclusion criteria were patients with positive dengue reverse-transcriptase polymerase chain reaction (PCR) [21] or probable dengue (WHO 1997 [11] or WHO 2009 [9]) with positive dengue IgM [22], [23].

An elderly patient referred to one whose age was 60 or greater [24]. A Charlson co-morbidity score was assigned for each patient based on the presence and severity of diseases listed in the index [25] and a Pitt bacteremia score validated against APACHE II score was calculated for each patient as previously described [26].

### Definitions

Leukopenia was defined as total white cell count (WCC) <4×10^9^/L for patients managed during 2005 and as total WCC <3.6×10^9^/L for patients managed from 2006 on-wards (laboratory reference range revised in 2006). Warning signs from the WHO 2009 guideline included: abdominal pain or tenderness, persistent vomiting, mucosal bleeding, clinical fluid accumulation, lethargy, hepatomegaly and rise in hematocrit (≥20%) concurrent with rapid platelet drop to <50×10^9^/L [9]. The diagnosis of DHF based on the WHO 1997 guidelines required the presence of fever, thrombocytopenia (platelet count <100×10^9^/L), bleeding (bleeding from the mucosa, gastrointestinal tract, injection sites or other locations) and plasma leakage (clinical fluid accumulation, hypoproteinemia, increase in hematocrit of ≥20% or decrease in hematocrit of ≥20% after fluid resuscitation) [11]. DSS was diagnosed in patients with either rapid and weak pulse with narrow pulse pressure (<20 mmHg) or hypotension in a patient with DHF [11]. SD defined according to the WHO 2009 guideline required one of the following criterion; severe plasma leakage, severe bleeding or severe organ involvement [9]. Severe plasma leakage was defined as either clinical fluid accumulation or hematocrit change of >20% in combination with at least one of the following; tachycardia (pulse >100/minute), hypotension (systolic BP <90 mmHg) or narrow pulse pressure (<20 mmHg). Severe bleeding was defined as hematemesis, melena, menorrhagia or drop in hemoglobin requiring transfusion of blood products. Severe organ involvement included hepatic injury (alanine aminotransferase >1000 U/L or aspartate aminotransferase >1000 U/L), impaired consciousness or myocarditis [9].

HAI was defined as infection acquired after two days of hospital admission [27]. Urinary tract infection (UTI) was defined as a positive urine culture with clinical features of UTI. Bloodstream infection was defined as a clinically significant bacterium isolated from blood culture. Pneumonia was defined by the presence of new consolidation on chest X-ray with compatible clinical signs and symptoms. *Clostridium difficile* infection was defined as positive stool *Clostridium difficile* toxin with diarrhoea.

### Statistical analysis

Outcome variables were categorized into dengue severity and poor clinical outcome. Dengue severity included patients with DHF, DSS and SD. Poor clinical outcome included patients who died, were admitted to the ICU or had excess length of stay (LOS). Excess LOS was defined as hospital admission greater than six days.

The chi-squared test and Fisher's exact test were used to compare univariate associations between categorical variables and the Mann-Whitney U test was used to compare continuous variables. A multiple logistic regression model based on inpatient data was built to ascertain how age was associated with excess LOS. The model adjusted for potential confounders including Pitt bacteremia score, HAI, Charlson co-morbidity score and dengue severity. The Hosmer-Lemeshow goodness-of-fit-test was applied to ensure the model fitted the data appropriately. A change in Pearson chi-squares graph was generated to identify the outlying and influential observations that may have affected the goodness-of-fit. Once identified the outlying and influential observations were tentatively removed and an auxiliary logistic regression model was rebuilt. The results of the two models were then compared in terms of the change in adjusted odds ratio (aOR) and their 95% confidence intervals (C.I.). All statistical analyses were performed using R version 2.15.2 and Stata 12.0 (Stata Corporation, Texas, U.S.A.). All tests were carried out with the 95% C.I. (equivalent to 5% ce level).

## Results

During the study period, of the 6989 dengue patients managed at CDC, 295 (4.3%) were elderly. There were 2034 (29%) who were PCR positive and 4955 (71%) who met the WHO criteria for probable dengue and were dengue IgM positive. There were a significantly higher proportion of PCR positive patients in the elderly (115/295, 39.1%) versus the adults (1919/6694, 28.7%).

Clinical, laboratory features and co-morbidities are shown in Table 1. Classical dengue symptoms of headache, rash and aches and pains were more common in the adults at presentation. Mucosal bleeding was significantly more common in the adults (24.2%) versus the elderly (12.5%) (p<0.001). Leukopenia at presentation was significantly more likely in the adults (5105/6694, 76.3%) versus the elderly (188/295, 63.7%) (p<0.001). The elderly were less likely to fulfill WHO 1997 probable dengue (93.6% versus 96.4%, p<0.001) as they were less likely than adults to have headache. In contrast, WHO 2009 probable dengue classification, which does not include headache as a criterion, was similar between the two groups (p = 0.167). Despite the atypical presentation of dengue in elderly patients there was no significant difference in time to dengue diagnosis between the two groups. The majority of patients (96%) were diagnosed on day one of admission with possible selection bias as the cohort was managed by the infectious diseases unit.

**Table 1:** Clinical characteristics of elderly patients (≥60) and adults (<60) with dengue fever.

Variable	Patient number (%)		P values
	Adults n = 6694	Elderly n = 295	
**PCR positive**	1919 (28.7)	115 (39.1)	0.001
**Parameters at presentation**			
Fever	6287 (93.9)	273 (92.5)	0.335
Malaise, lethargy	1886 (28.2)	100(33.9)	0.033
Rash	3186 (47.6)	108 (36.6)	<0.001
Headache	3287 (49.1)	104 (35.3)	<0.001
Any aches and pains	5495 (82.1)	234 (79.3)	0.226
Nausea	3753 (56.1)	144 (48.8)	0.014
Vomiting	2856 (42.7)	113 (38.3)	0.138
Mucosal bleeding	1620 (24.2)	37 (12.5)	<0.001
Hematuria	44 (0.7)	5 (1.7)	0.054
Melena	4 (1.2)	40 (0.6)	0.128
Leukopenia	5105 (76.3)	188 (63.7)	<0.001
**Fever duration (days)**	5 (3–7)	4 (2–7)	<0.001
**WHO classification**			
Probable dengue 1997	6450 (96.4)	276 (93.6)	0.014
Probable dengue 2009	4798 (71.5)	222 (75.3)	0.167
**Co-morbidities**			
Diabetes	126 (1.9)	64 (21.7)	<0.001
Hypertension	315 (4.7)	157 (53.2)	<0.001
Renal failure	20 (0.3)	12 (4.1)	<0.001
Malignancy	13 (0.2)	4 (1.4)	0.005
Immunocompromised	3 (0.1)	1 (0.3)	0.158
COPD	3 (0.1)	2 (0.7)	0.016
Charlsons co-morbidity score >3	7 (0.1)	8 (2.7)	<0.001
Pitt bacteremia Score ≥4	50 (0.7)	1 (0.3)	0.420
**Warning signs during clinical illness**			
Presence of any warning signs	4078 (60.9)	180 (61.0)	0.973
Malaise/lethargy	1886 (28.2)	100 (33.9)	0.033
Abdominal pain	1539 (30.0)	66 (22.4)	0.805
Mucosal bleeding	1620 (24.2)	37 (12.5)	<0.001
Hematocrit rise with rapid platelet drop	397 (5.9)	25 (8.5)	0.073
Hepatomegaly	70 (1.0)	9 (3.1)	0.006
Clinical fluid accumulation	37 (0.6)	4 (1.4)	0.093
Persistent vomiting	0 (0)	0 (0)	NA

Abbreviations: PCR = polymerase chain reaction, WHO = World Health Organization, COPD = chronic obstructive pulmonary disease.

The elderly were significantly more likely to have co-morbidities (Table 1) including hypertension, diabetes, chronic renal impairment and chronic obstructive pulmonary disease. As expected a high Charlson co-morbidity score (>3) was significantly more common in elderly patients (p<0.001) reflecting a higher burden of co-morbidities.

Overall there was no significant difference in the frequency of warning signs between the two groups. However when individual warning signs were analyzed the elderly were more likely to have hepatomegaly (p = 0.006) and malaise/lethargy (p = 0.033) while the adults had significantly more mucosal bleeding (p<0.001).

The elderly were more likely to require hospitalization (265/295, 89.8%) versus the adults (5363/6694, 80.1%) (p<0.001), this is expected as the admission criteria from March 2007 included elderly patients with co-morbidities. Clinical outcomes are shown in Table 2. DHF occurred significantly more in the elderly cohort (86/295, 29.2%) versus the adults (1431/6694, 21.4%) (p = 0.002). Likewise, SD was more likely in the elderly (60/295, 20.3%) versus their younger counterparts (975/6694, 14.6%) (p = 0.006). Despite more severe disease in the elderly, ICU admission and death were not significantly different between elderly and adult dengue patients.

**Table 2:** Outcomes for elderly (≥60) and adult (<60) patients with dengue fever.

Variable	Patient number (%)		P values
	Adults n = 6694	Elderly n = 295	
**Dengue severity**			
DHF	1431 (21.4)	86 (29.2)	0.002
DHF Grade I–II	1199 (17.9)	80 (27.1)	<0.001
DSS	232 (3.5)	6 (2.0)	0.184
SD	975 (14.6)	60 (20.3)	0.006
**SD criteria**			
Severe bleeding	401 (41.1)	13 (21.7)	0.003
Severe plasma leakage	332 (34.1)	17 (28.3)	0.363
Severe organ involvement	118 (12.1)	12 (20)	0.100
SB+SPL	67 (6.9)	3 (5.0)	0.792
SB+SOI	14 (1.4)	1 (1.7)	0.594
SPL+SOI	30 (3.1)	10 (16.7)	<0.001
SB+SPL+SOI	13 (1.3)	4 (6.7)	0.014
**Outcome**			
ICU	13 (0.2)	2 (0.7)	0.130
Death	3 (0.1)	0 (0)	1
**HAI**			
Any HAI	66 (1.2)	13 (4.9)	<0.001
Pneumonia	36 (0.7)	10 (3.8)	<0.001
UTI	17 (0.3)	5 (1.9)	0.003
*Clostridium difficile*	1 (0)	0 (0)	1
Bloodstream infection	14 (0.3)	0 (0)	1

Abbreviations: DHF = dengue haemorrhagic fever, DSS = dengue shock syndrome, SD = severe dengue, SB = severe bleeding, SPL = severe plasma leakage, SOI = severe organ involvement, ICU = intensive care unit, HAI = hospital acquired infection, UTI = urinary tract infection.

LOS was longer in the elderly with a median of five days versus four days in adults. HAI occurred at greater frequency in the elderly (13/295, 4.9%) versus the adults (66/6694, 1.2%). Notably pneumonia and UTI were the most common HAIs. There were no episodes of bloodstream infection in the elderly versus 14 episodes in the adults, but this did not reach significance. *Clostridium difficile* infection was detected in one adult patient. Older people were more likely to be admitted longer, after adjusting for HAI, Charlson score, Pitt bacteremia score and dengue severity (Table 3).

**Table 3:** Excess length of hospital stay.

Variable	Adjusted odds ratio*	95% Confidence interval
**Elderly (age ≥60)**		
No (n = 5774)	Reference	Reference
Yes (n = 296)	2.01	1.37–2.88
**Critically ill**		
Pitt bacteremia score <4 (n = 6016)	Reference	Reference
Pitt bacteremia score ≥4 (n = 54)	5.13	2.59–9.75
**Hospital-acquired infection**		
No (n = 5988)	Reference	Reference
Yes (n = 82)	12.06	7.39–19.90
**Charlsons co-morbidity score**		
≤3 (n = 6054)	Reference	Reference
>3 (n = 16)	6.90	2.02–22.56
∧ **Dengue Severity**		
Not severe (n = 3840)	Reference	Reference
Severe (n = 2230)	2.24	1.83–2.74

*All p-values <0.001.

∧ Dengue Severity – dengue hemorrhagic fever, dengue shock syndrome or severe dengue.

## Discussion

Dengue is a neglected tropical disease that is increasingly affecting elderly patients. As the dengue epidemic evolves and the population ages dengue in the elderly is likely to be commonplace. Diagnosis in this group may be challenging as the presentation can be atypical. Delayed diagnosis may delay lifesaving interventions. Elderly patients have worse outcomes compared with younger counterparts with increased rates of DHF, SD and dengue-related mortality. Elderly patients have higher rates of HAI placing them at risk of infection-related mortality. Elderly patients have an increased length of hospitalization as a result of severe disease, co-morbidity and HAI. This will place further burden on already stretched hospital systems.

Elderly patients had worse clinical outcomes with significantly higher rates of DHF and SD as previously reported [10], [17]. In our study this did not result in increased mortality unlike in Puerto Rico [17] and Taiwan [7], [18]. The reasons for this are unclear as the elderly cohort suffered more severe disease. The common manifestations of SD in the elderly were severe plasma leakage and severe bleeding. Despite more severe disease in the elderly, they did not experience more warning signs. In adult patients with confirmed dengue no single warning sign was highly sensitive in predicting either DHF or SD [28]. However hepatomegaly, persistent vomiting, hematocrit rise concurrent with rapid platelet drop and clinical fluid accumulation are highly specific for the development of both DHF and SD [28]. In our study hepatomegaly occurred significantly more commonly in the elderly. Vigilance for the above warning signs in the elderly is warranted to ensure rapid provision of potentially lifesaving interventions, while recognising the limitations of warning signs so as not to be falsely reassured in their absence.

DHF characterized by plasma leakage and bleeding is more common in secondary dengue infections [29]. In Singapore, older age is a significant risk factor for past dengue infection [30]. In a seroepidemiologic study of adults, 88.9% aged 55–74 years had evidence of past infection compared with 17.2% of young adults (18–24 years) [30]. Based on this data it is likely that many patients in our elderly cohort had secondary dengue infections increasing their risk of DHF [18].

Higher rates of DHF in the elderly may be the result of co-morbidities. In our cohort, elderly patients were significantly more likely to have hypertension and diabetes, both of which were recognised as risk factors for DHF [31]. A case-control study of DHF and dengue patients in Singapore demonstrated that adult patients with concomitant diabetes and hypertension were at higher risk of DHF, compared with patients without these co-morbidities [31]. The pathogenesis underlying this relationship is unclear but diabetes mellitus results in immune dysfunction [32] in addition to concomitant immunosenescence [13]. Elderly dengue patients with diabetes should be admitted for close monitoring, as close monitoring and early intervention with fluid therapy maybe lifesaving.

Concurrent infection has been reported in patients with dengue fever, including malaria, leptospirosis and *Staphylococcus aureus* [33], [34], [35]. Acute dengue infection can impair T-cell proliferation in vitro suggesting that dengue may modulate the immune system increasing susceptibility to co-infection [36]. Likewise, age results in immune dysregulation with defects in T and B cell function and impaired cytokine response [13]. Dengue and immunosenescence could explain the increased rates of HAI in elderly patients in our cohort. Median time from illness onset to death in dengue patients in Singapore was 12 days suggesting that HAI may have contributed [8]. Bacteremia was documented in 14.3% of the deaths with a median duration of 6.5 days from admission [8]. Prolonged fever (>5 days) and ARF were independent predictors of concurrent bacteremia in DHF patients in Taiwan [37]. Leukocytosis was more common in patients with dual infection versus controls but did not reach significance in this small cohort [37]. In our cohort neutrophilia was associated with nosocomial infection and excess LOS. Clinicians need to be aware of the potential for bacterial co-infection in elderly patients as they are at higher risk of mortality from severe sepsis versus younger counterparts [13], [38].

There are limitations to our retrospective study. Firstly, HAI may have been under reported as patients could have received empiric treatment without clinical investigation. Secondly, the risk of HAI can be increased by the presence of invasive devices such as intravenous and urinary catheters. The number of patients in this study who had invasive devices prior to the onset of HAI is unknown. Thirdly, the study was conducted at a single medical centre so the severity of illness may be biased by referral pattern. Future studies should include elderly patients managed in the community to enhance our understanding of the factors that affect hospital admission and outcome.

Dengue in the elderly is an emerging phenomena and remains incompletely understood. Dengue should be considered in the differential diagnosis of fever in elderly patients with appropriate epidemiological exposure, and diagnostic testing should be considered as this group presents atypically. Early diagnosis is critical for appropriate monitoring. Elderly patients are at higher risk of DHF and SD, especially those with pre-existing co-morbidities. A lower threshold for hospital admission may be required in this group for close monitoring. Clinicians need to remain vigilant for HAI in elderly dengue patients as these occur at increased frequency and may confer mortality risk.

## Supporting Information

Checklist S1 STROBE checklist.
(DOC)
