# Correction: Challenges in Dengue Fever in the Elderly: Atypical Presentation and Risk of Severe Dengue and Hospita-Acquired Infection

**DOI:** 10.1371/journal.pntd.0002886

**Published:** 2014-04-25

**Authors:** The PLOS Neglected Tropical Diseases Staff

The word “Hospital-Acquired” is misspelled in the article title. The correct title is: Challenges in Dengue Fever in the Elderly: Atypical Presentation and Risk of Severe Dengue and Hospital-Acquired Infection**.** The correct citation is: Rowe EK, Leo Y-S, Wong JGX, Thein T-L, Gan VC, et al. (2014) Challenges in Dengue Fever in the Elderly: Atypical Presentation and Risk of Severe Dengue and Hospital-Acquired Infection. PLoS Negl Trop Dis 8(4): e2777. doi:10.1371/journal.pntd.0002777
